# Polarization-driven thermal emission regulator based on self-aligned GST nanocolumns

**DOI:** 10.1016/j.isci.2022.105780

**Published:** 2022-12-09

**Authors:** Joo Hwan Ko, Do Hyeon Kim, Sung-Hoon Hong, Sun-Kyung Kim, Young Min Song

**Affiliations:** 1 School of Electrical Engineering and Computer Science, Gwangju Institute of Science and Technology, Gwangju 61005, Republic of Korea; 2 ICT Materials and Components Research Laboratory, Electronics and Telecommunications Research Institute (ETRI), Daejeon 34129, Republic of Korea; 3 Department of Applied Physics, Kyung Hee University, Gyeonggi-do, Yongin-si 17104, Republic of Korea; 4 Anti-Viral Research Center, Gwangju Institute of Science and Technology, Gwangju 61005, Republic of Korea; 5 AI Graduate School, Gwangju Institute of Science and Technology, Gwangju 61005, Republic of Korea

**Keywords:** Nanotechnology, Thermal engineering, Thermal property

## Abstract

The increasing advances in thermal radiation regulators have attracted growing interest, particularly in infrared sources, thermal management, and camouflage. Despite many advances in dynamic thermal emitters with great controllability, sustained external energy is required to maintain the desired emission. In this study, we present a polarization-driven thermal emission regulator based on a two-way control: i) phase change and ii) polarization tuning. Based on a conventional, non-volatile phase change material, *i*.*e*., Ge_2_Sb_2_Te_5_ (GST), we newly introduce an anisotropic medium for facile emissivity regulation without heat energy consumption. A rigorous coupled-wave analysis method provides design guidelines for finding optimal structural parameters. We utilized a simple glancing angle deposition process which induces tilted self-aligned nanocolumns with anisotropic properties. The fabricated sample shows polarization-sensitive thermal regulation through thermal imaging spectroscopic measurement. Additionally, we manufactured a multispectral visibly/thermally camouflaged patch that identifies encrypted information at a specific polarization state for a proof-of-concept demonstration.

## Highlights

• Finely tunable thermal emitter through control of phase and linear polarization state
• Scalable fabrication of self-aligned GST nanocolumns with anisotropic geometry
• Active thermal camouflage with multispectral optical encoding function
• Robust angular dependency assuring hemispherical emission performance

## Graphical abstract

**Figure:**
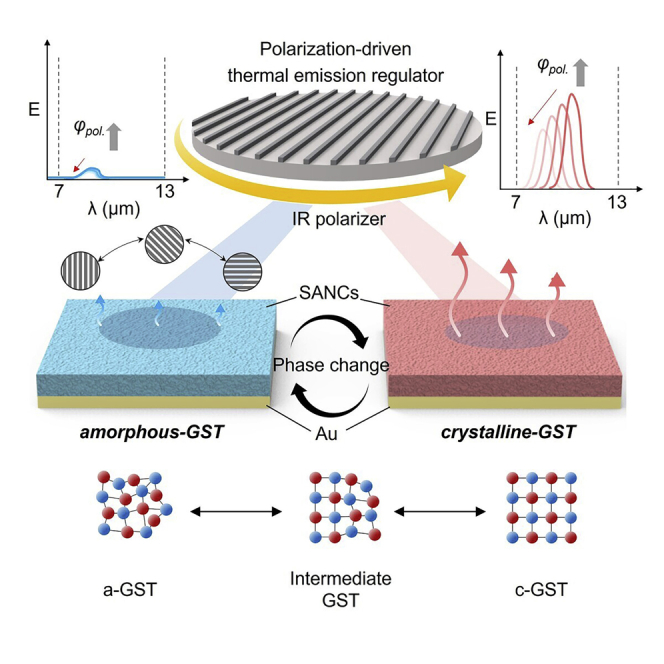

## Introduction

Perpetual progress in functional surfaces that control thermal emissions across the electromagnetic spectrum has promoted comprehensive implications for fundamental science and future applications, including adaptive thermal management, *e*.*g*., radiative cooling,^1^^,^^2^^,^^3^^,^^4^^,^^5^^,^^6^ thermal infrared sources,^7^^,^^8^^,^^9^ and active thermal camouflage.^10^^,^^11^^,^^12^^,^^13^^,^^14^^,^^15^^,^^16^ According to the Stefan–Boltzmann law, all objects with a temperature higher than absolute zero spontaneously radiate electromagnetic energy, known as thermal radiation.^17^ In addition, at thermodynamic equilibrium, according to Kirchhoff’s radiation law, thermal emissivity refers to the wavelength-specific optical absorption of an object.^18^ Thus, the dynamic control of infrared emission requires spectral regulation based on the modulation of light–matter interactions. To achieve the dynamic response from the initial structures, material property and/or structural parameter should be controlled (*e*.*g*., physical dimension, optical property, electron density, or band structure). Over the last decades, innovative approaches enable dynamic response to light–matter interactions, including 1) electrical control of the intersubband absorption of quantum wells^19^^,^^20^ and 2) mechanical modulation of the distance between the metamaterial and metal film.^21^^,^^22^ Despite the excellent reversibility and controllability of these methods, low energy input and wide tuning range are still the key prerequisites to be overcome.

One class of promising materials that have shown great attention for tunable photonics is the phase change material (PCM) based on large complex refractive index variation, resulting in a significant emissivity change.^23^^,^^24^^,^^25^^,^^26^ Modulation of the optical properties of PCMs, such as VO_2_ and Ge-Sb-Te (GST), occurs in both the real and imaginary parts that result from changes in the crystal structure or atomic bond configuration following the metal–insulator transition (MIT).^27^ Various photonic structures have been introduced based on the VO_2_, including multilayered structures,^4^^,^^28^^,^^29^^,^^30^ two-dimensional grating,^1^^,^^31^^,^^32^^,^^33^ and gradient films,^34^ resulting in excellent thermal emissivity control. However, a volatile characteristic of VO_2_ at the transition temperature requires sustained external energy to maintain the desired emission. On the other hand, chalcogenide PCM (*e*.*g*., GST, Sb_2_S_3_, and In_3_SbTe_2_) exhibits non-volatile modulation properties, enabling their utilization as non-volatile nanostructured antennas,^35^^,^^36^ heat radiating devices,^37^ radiative coolers,^38^ and thermal camouflage with large variations in thermal emission.^39^^,^^40^^,^^41^ Based on the Lorentz–Lorenz relation, the effective refractive index of the intermediate phases of GST can be estimated. Thus, Qu et al. presented a continuously controllable thermal emitter between crystalline GST (c-GST) and amorphous GST (a-GST).^40^ The inherent phase change process, which modifies the atomic bonding configuration at two different temperatures ⅰ) crystallization temperature (T_c_ ≈ 160°C) and ⅱ) melting temperature (T_m_ ≈ 600°C, with the quenching process), not only hinders precise control of the targeted intermediate phase but also demands large energy consumption.

Here, we newly present a polarization-sensitive GST medium for facile thermal-emission regulation without heat energy consumption. As an efficacious approach, scalable self-aligned nanocolumns (SANCs) are introduced, composed of GST with anisotropic geometry through glancing angle deposition (GLAD).^42^^,^^43^ By designing an optical structure with a stacking configuration (*i*.*e*., an anisotropic GST medium on a metal reflector), the selective resonance elaborately varies according to the polarization state, resulting in fine tunability. Furthermore, target emissivity is realized apart from the continuous heat energy by adjusting the polarization angle within the modulation range. For practical demonstration with selective emissivity control, the multispectrally hidden patch is designed to hide the pattern at normal conditions; inversely, it reveals the hidden information at the specific polarization condition for an encrypted peer identification system. In the design process, we introduced a computational model to optimize the resonance characteristics according to both variables (*i*.*e*., phase of GST and polarization state) to maximize the thermal emission variation. Furthermore, rigorous coupled-wave analysis is performed by considering various parametric combinations.

### Thermal emission regulation using polarization-sensitive nanocolumns

**Figure 1:**
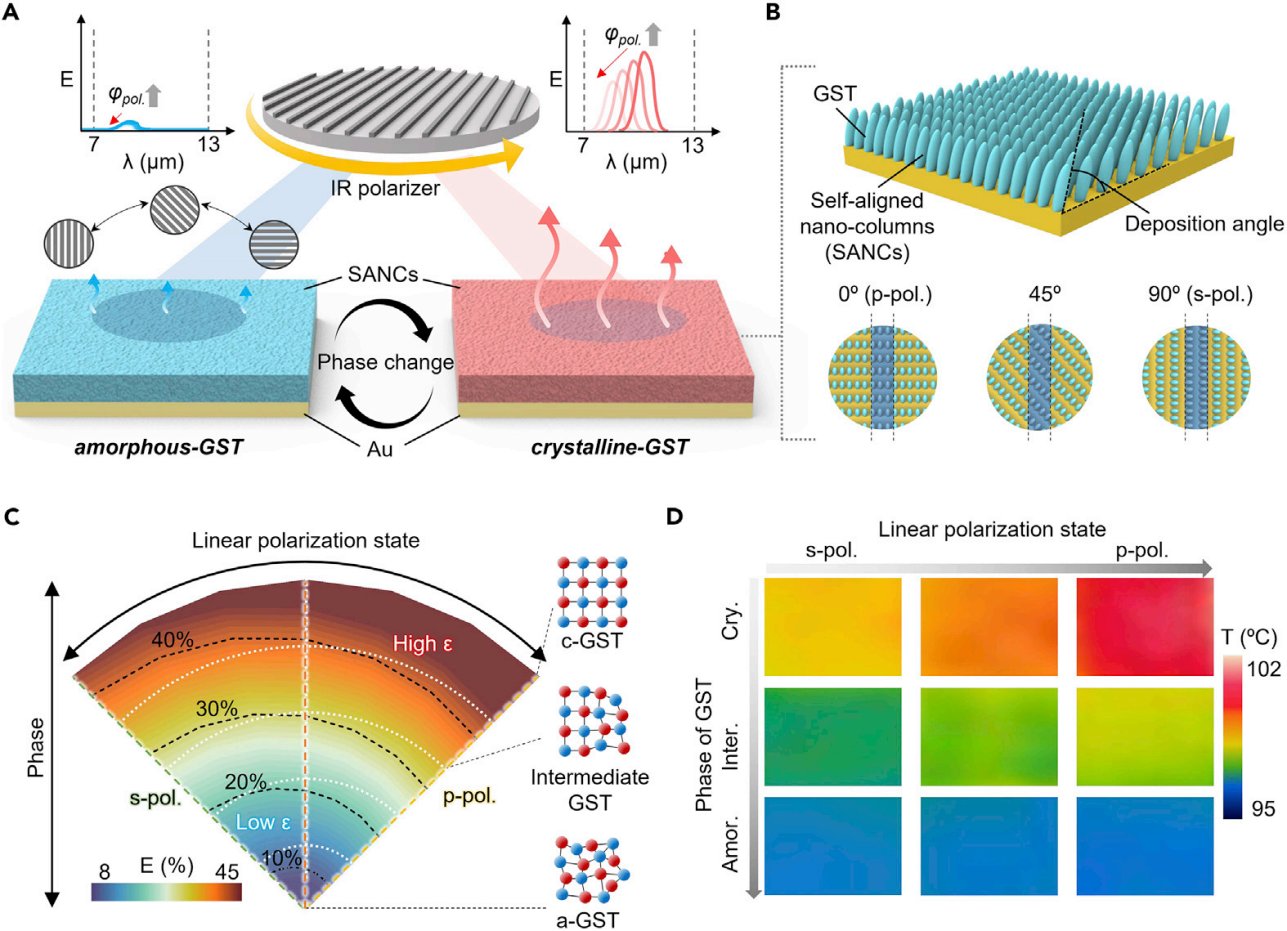
Overview of polarization-sensitive thermal emitter based on self-aligned Ge_2_Sb_2_Te_5_ nanocolumns (A) Schematic of a polarization switchable thermal emitter using self-aligned nanocolumns (SANCs). (B) Illustration of SANCs with top views according to different polarization angles. (C) Emissivity variation diagram of SANCs with respect to polarization states and phases. (D) Thermal images of fabricated SANCs at different polarization angles (0°, 45°, and 90°) and phases (crystalline, intermediate, and amorphous).

Figure 1A shows a schematic of a polarization-driven thermal emission regulator consisting of self-aligned GST nanocolumns and a gold substrate. The emissivity of SANCs varies with the change in polarization. Because c-GST exhibits a higher extinction coefficient than a-GST, it features a strong resonance; accordingly, the deviation in emissivity is higher for c-GST than for a-GST (Figure S1). The polarization-sensitive characteristic originates from the geometrical difference in the plane orientation (Figure 1B). Owing to the higher porosity at s-polarization (*i*.*e*., slanted cross-section of SANCs), the effective complex refractive index is lower than that of the polarization state with p-pol (*i*.*e*., a vertical cross-section of SANCs). The SANCs were slanted by tilting the substrate in the direction of the incoming vapor at a specific deposition angle. Owing to the atomic shadowing effect during the deposition process, the porosity of the SANCs varies with the view of the plane, which results in polarization-dependent properties. For a high emissivity contrast, we deposited SANCs at a deposition angle of 70°, where a larger optical constant difference is achieved for each polarization (*i*.*e*., p-polarization and s-polarization).^42^ Figure 1C shows the emissivity variation diagram of the proposed thermal regulator with respect to the phase and polarization states. The emissivity of SANCs changes with the phase transition which is generally known as a unique property of GST, but SANCs also regulate the emissivity by controlling the polarization angle (black dashed lines). Thus, introducing polarization to SANCs facilitates infrared emission modulation without a heating source, whereas conventional thermal emitters using PCM require external heat energy to tune the emissivity. Furthermore, the modulation of polarization angles broadens the emissivity range beyond that of isotropic GST. The effective refractive index and emissivity spectra for different GST crystallization fractions are presented in Figures S2 and S3, respectively. To examine emittance switching, we performed thermal infrared imaging of the entire sample (crystalline, intermediate, and amorphous) at different polarization angles (Figure 1D). The SANCs exhibited a marked change of thermal infrared temperature in the polarization angle variation. This difference decreases with the phase transition from crystalline to amorphous owing to low emissivity. These measurement results demonstrate the fine tunability of SANCs over polarization change, exhibiting well-matched results with the calculations shown in Figure 1C.

### Mechanism of ultrathin film interference and spectral characteristics

**Figure 2:**
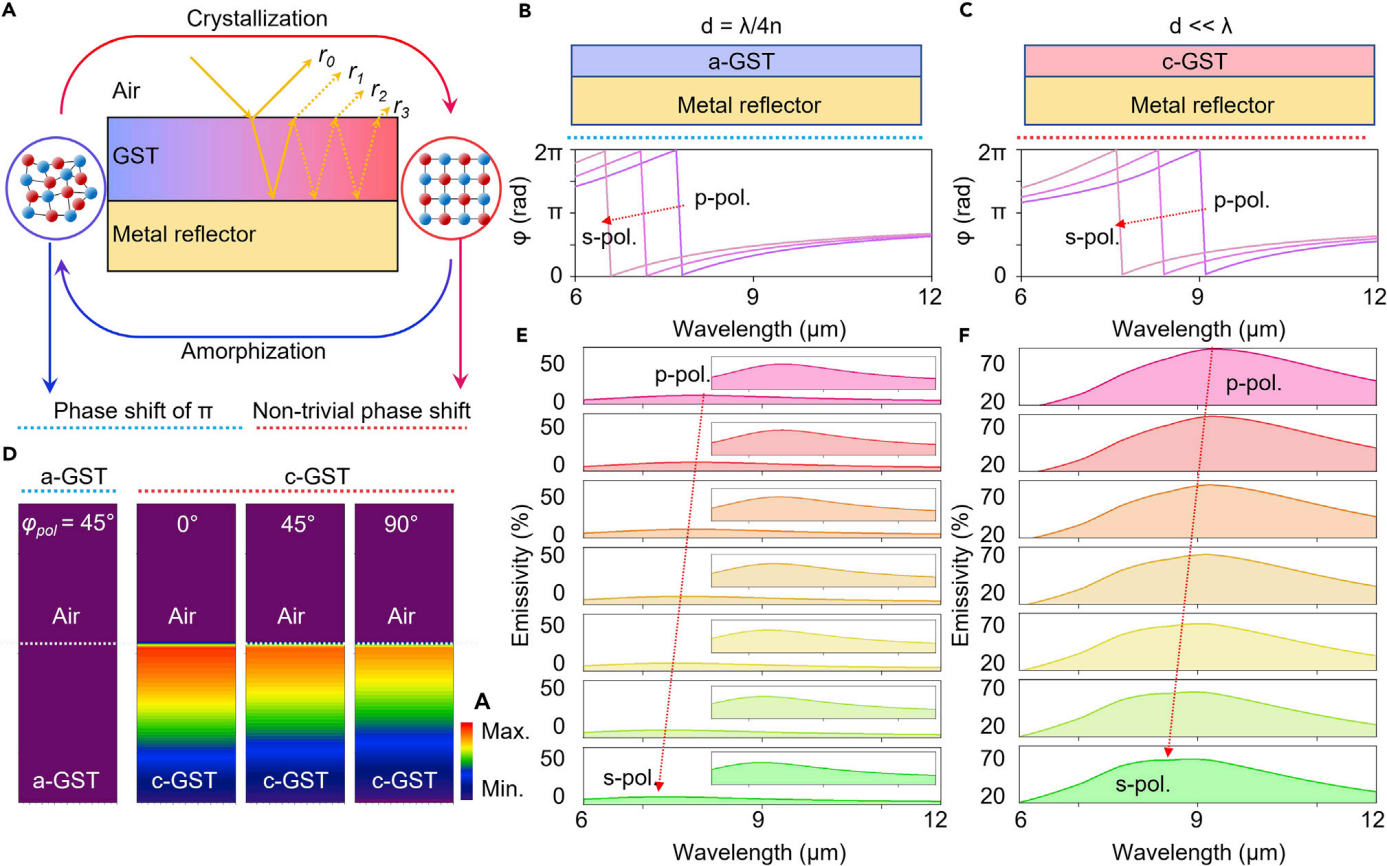
Influence of thin film losses and absorption behavior (A) Destructive interference of light reflection on the stacked layers comprised of GST and metal reflector. Corresponding to the phase of GST, the resonance mode refers to the phase shift of π or non-trivial phase shift. (B) Schematic of calculation structure composed of SANCs (a-GST) on Au with quarter-wavelength thickness and reflection phase shift with polarization angle. (C) Schematic of calculation structure composed of SANCs (c-GST) on Au with non-trivial phase shift and reflection phase shift with polarization angle. (D) Absorption intensity distribution of SANCs with the metal reflector in each phase of GST and polarization angle. (E) Simulated emissivity spectra corresponding to the polarization angle change of SANCs (a-GST) on Au. Each inset represents closed reflectance spectrum with the intensity range of 0%–10%. (F) Simulated emissivity spectra corresponding to the polarization angle change of SANCs (c-GST) on Au.

To achieve large emissivity variation by both the phase of GST and polarization, we optimized the geometrical parameters (*i*.*e*., thickness and porosity of GST). First, as a basic structure, Figure 2A shows a schematic of a GST thin film lying on a metallic mirror. The reflection coefficient (*r*) of the system can be expressed as

$$r=\frac{r_{12}+r_{23}e^{2i\beta }}{1+r_{12}r_{23}e^{2i\beta }}\text{,}$$

where *r*_*pq*_ = $\left({\tilde{n}}_{p}-{\tilde{n}}_{q}\right)/\left({\tilde{n}}_{p}+{\tilde{n}}_{q}\right),{\tilde{n}}_{p}=n_{p}+{ik}_{p}$ and $\beta =\left(2\pi /\lambda \right){\tilde{n}}_{2}h$.^44^ For an a-GST thin film with negligible loss, the interface phase shifts are either 0 or π; thus, the resonance condition is determined by the phase accumulation at a wavelength corresponding to the optical thickness of the quarter wavelength, d = λ/4n (Figure 2B). Therefore, the phase value shows that the resonance condition shifts corresponding to the polarization change owing to refractive index modulation. Whereas, as represented in Figure 2C, in the case of the c-GST thin film, a lossy condition causes the reflection phase to differ significantly from π with a complex phase shift, resulting in a strong resonance condition while canceling out r_0_, even when d ≪ λ.^45^^,^^46^ Hence, the phase graph shows that the resonance wavelength shifts following the polarization change corresponding to the complex refractive index variation. Optical behavior was confirmed by optical simulations of the absorption profile of the GST layer under various conditions (*i*.*e*., a-GST/c-GST and s-polarization/p-polarization). Figure 2D shows the simulation results, indicating that the a-GST/Au structure shows negligible absorption, whereas c-GST/Au shows strong absorption at the c-GST layer with high polarization sensitivity. Consequently, to comprehensively confirm the thermal emission properties in terms of both performance and tunability, we calculated the absorption spectra of a-GST/Au and c-GST/Au at different polarization angles. As expected from the resonance mechanism, the absence of the extinction coefficient of a-GST caused an extremely low absorption (under 10% at the resonance wavelength, Figure 1E). In contrast, the presence of the extinction coefficient of c-GST strengthened the resonance intensity (Figure 2F). Notably, the resonance wavelength shifts originating from the effective complex refractive index change corresponding to each polarization state for both cases that implies that emissivity tuning over all intermediate states is possible.

### Tuning range of emitted power with SANCs thickness variation

**Figure 3:**
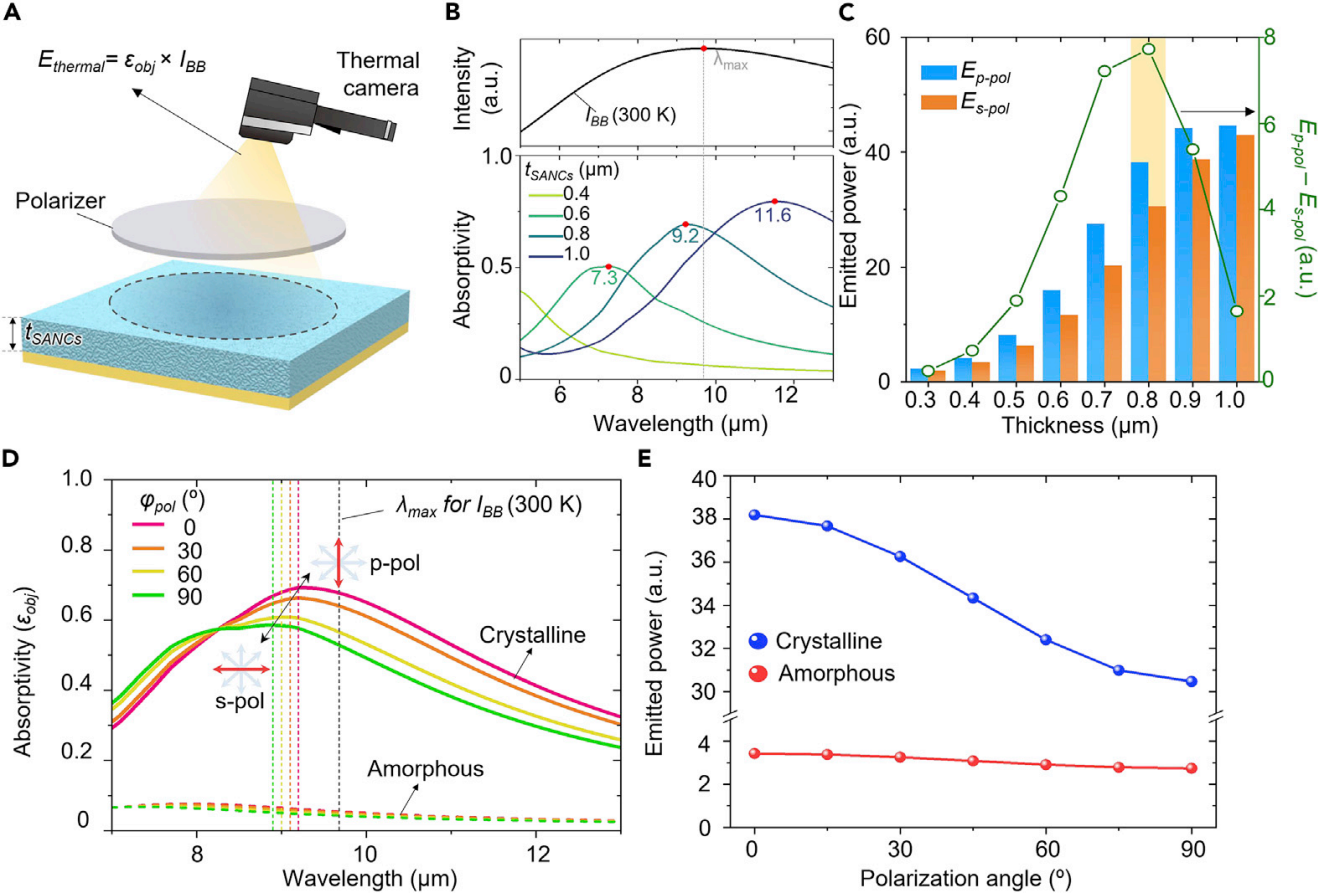
Emitted power with respect to SANCs thickness and polarization angle (A) Schematic of a thermal imaging demonstration of SANCs with a polarizer. (B) Calculated blackbody radiation curve at 300 K (top) and emissivity spectra for different SANCs thicknesses (bottom). (C) Evaluated emitted power of SANCs under p-polarized and s-polarized lights. The green lines present the difference of emitter power between both polarization states. (D) Calculated emissivity spectra of optimized SANCs (*t*_*SANCs*_: 0.8 μm) with different polarization angles and phase. The short dashed lines indicate the wavelengths of emission peak. (E) Calculated emitted power of optimized SANCs as a function of polarization angle.

The emissivity tuning of the SANCs by polarization can be demonstrated by thermal infrared imaging (Figure 3A). The thermal camera measures the emitted power of the object by integrating the emissivity and blackbody spectral radiance over the spectral range of the thermal infrared camera from 7 to 13 μm.^47^ Detailed information on the emitted power equation is described in STAR Methods. As the peak wavelength (*λ*_*max*_) of the blackbody (∼300 K) radiation curve is at 9.7 μm (Figure 3B; top), the emitted power increases when the peak of emissivity is close to *λ*_*max*_ (Figure S3). To extend the tuning range of the emitted power (*P*_*p-pol*_ – *P*_*s-pol*_), the difference between the emitted power for p-polarized light and s-polarized light, the peak wavelength of emissivity should be close to the maximum point of blackbody radiation for the p-polarization state. As shown in Figure 3B, the emission peak at a thickness of 0.8 μm is the closest to the peak wavelength of blackbody spectral radiance. The tuning range of the emitted power as a function of SANCs thickness (*t*_*SANCs*_) is presented in Figure 3C. The emitted power difference is the largest at a thickness of 0.8 μm because this condition exhibits the point closest to the peak wavelength, as shown in Figure 3B. The difference in emitter power decreases above the thickness of 0.8 μm as the thicker SANCs increase the emitted power for s-polarization owing to the emissivity peak being close to the blackbody radiation peak (Figure S4). Thus, a thickness of 0.8 μm is selected for optimization that satisfies the condition of broadening the tuning range.

Figure 3D shows the calculated emissivity spectra of the optimized SANCs in the crystalline and amorphous states as a function of the polarization angle. The emissivity peak of the crystalline phase shifts linearly to a short wavelength with an increase in the polarization angle, whereas the emissivities of the amorphous phase are insensitive to the polarization angles because of the lower optical constants of the amorphous phase (Figure S1). Consequently, the tuning range of the emitted power over the polarization variation in the crystalline phase is broader than that of the amorphous phase (Figure 3E). The emitted power of SANCs decreases by ∼15% with the blue-shift of emissivity peak (∼0.4 μm), indicating the polarization angle variation from 0° to 90°. The variations in the emitted power for different crystalline fractions are shown in Figure S5. These results exhibit high emissivity differences for each polarization, indicating that the heat management of the SANCs can be observed using a thermal imager. The ultrathin structure of the SANC shows the robust angular dependency of the emissivity (Figure S6, for detail performance). Since thermal radiation is emitted in total hemispheric direction, the thermal emitted power was hemispherically integrated, resulting in similar tendency (see Figure S7 and STAR Methods for detail result).

### Fabrication of porous GST and experimental confirmation

**Figure 4:**
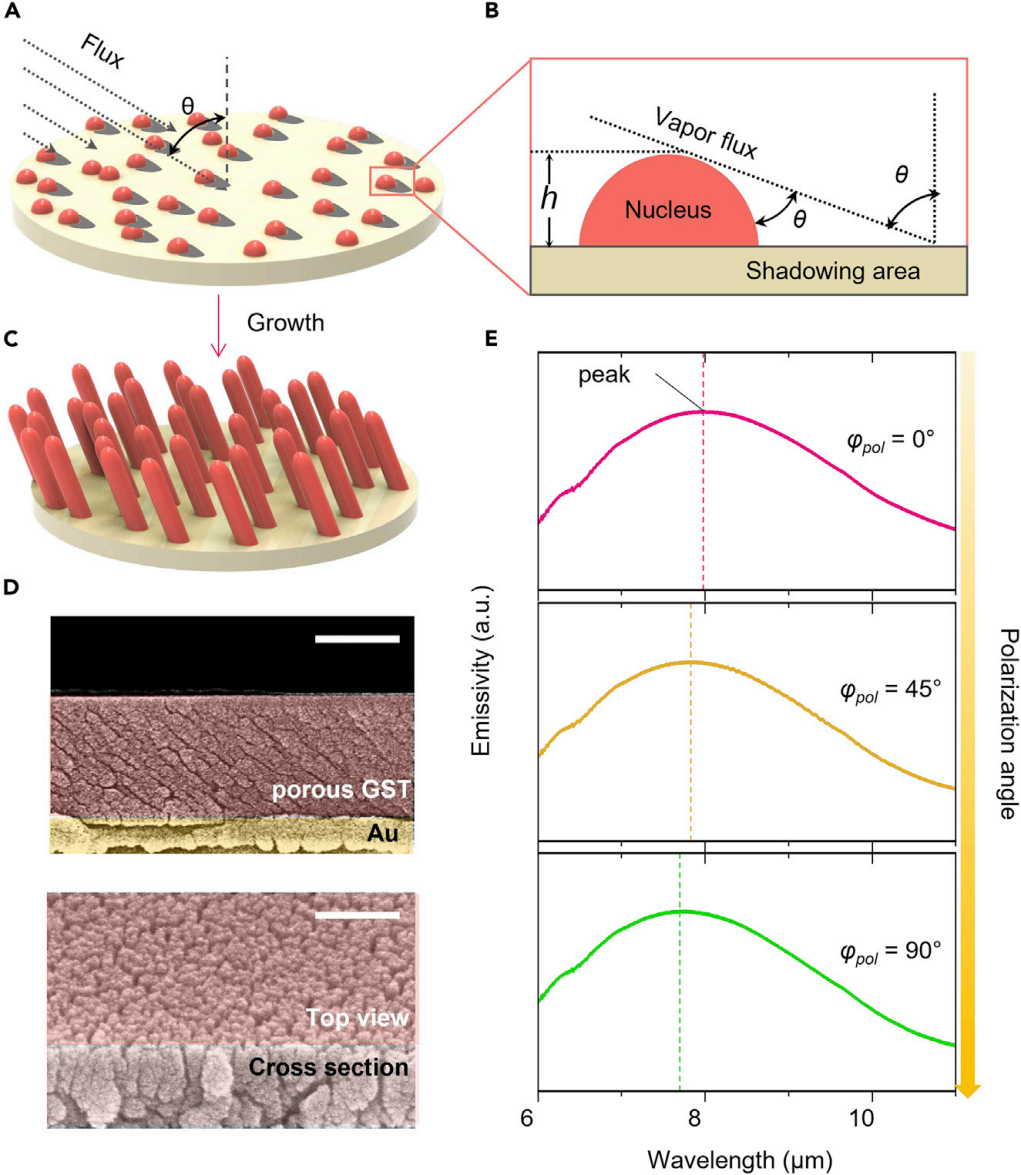
Experimental confirmation of self-aligned nanocolumns and optical behavior (A) Schematic of initial formation (nucleus) with the vapor flux angle (*θ*). (B) Closed view of self-shadow effect by glancing angle deposition with the geometrical parameters (height (*h*) and deposition angle (*θ*). (C) Schematic of SANCs after growth. (D) Cross-sectional scanning electron microscope (SEM) image (top), and tilted SEM image (bottom) of fabricated GST-SANCs on Au layer. Scale bar is 500 (top) and 100 nm (bottom). (E) Measured emissivity corresponding to the polarization angle.

To realize the designed SANCs experimentally, we modulated the deposition angle by applying nanopores between the nanocolumns using GLAD. As depicted in Figure 4A, the vapor flux that has an incident angle (*θ*) with respect to the Au substrate forms a porous and anisotropic geometry.^48^ At the initial stage of deposition, the evaporated particles form randomly distributed nanosized nuclei at the surface of the substrate. As shown in Figure 1B, the initial nucleated islands provide a self-atomic shadow effect by blocking the incident vapor flux, where the evaporated particles can no longer deposit. As schematically expressed, the shadowing length (*l*) depends on both the deposition angle and height of the nucleus (*h*), which is given by l=htanθ.^48^ As the deposition proceeded, the blocked atomic flow caused a self-shaded region, resulting in preferential deposition of the evaporated flux following the nucleus sites. Finally, anisotropic porous nanocolumns were obtained (Figure 4C). At deposition angles above 80°, the self-shadowing effect is sharply reduced that destroys the anisotropic property.^49^ Therefore, in this study, we selected the deposition angle of 75° for the largest anisotropic geometry. Figure 4D shows the fabricated SANCs with a cross-sectional/tilted view of a scanning electron microscopy image deposited on an Au substrate with a deposition angle of 75° without rotation. Figure 4E shows the measured emissivity spectra for SANCs on the Au reflector with changing polarization angle from s-polarization to p-polarization of the incident light. This result confirms that the fabricated sample shifts toward a shorter wavelength of ∼0.4 μm with increasing polarization angles, consistent with calculated results.

### Adaptive camouflage patch with encrypted pattern

**Figure 5:**
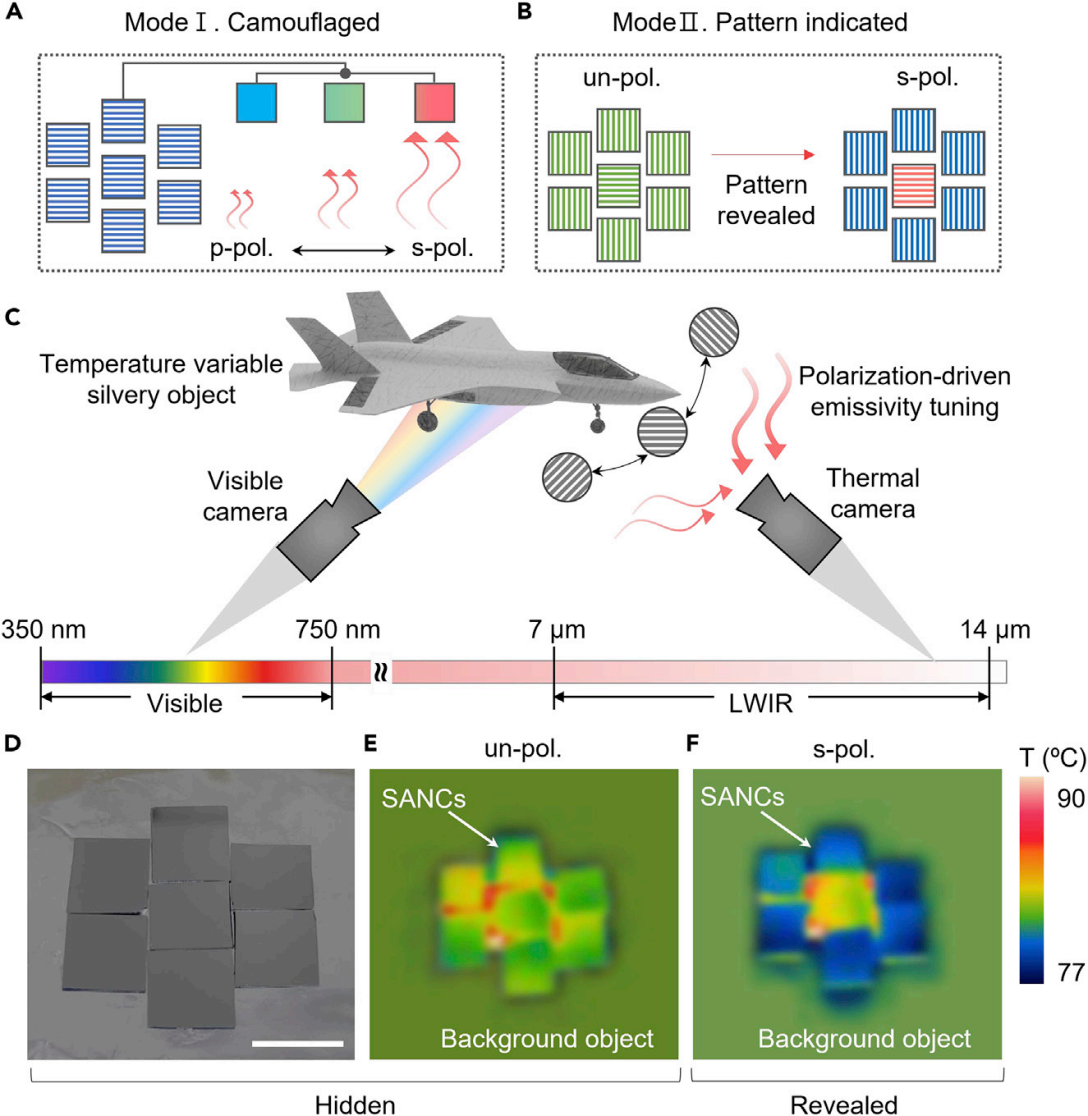
Conceptual demonstration of active thermal camouflage patch (A) Schematic of operation principle for active thermal camouflage patch. Mode Ⅰ is emissivity matching by modulating polarization state for arranged sample in the same direction. (B) Schematic of operation principle for decryption of hidden pattern (Mode Ⅱ) by polarization angle change by pre-arrangement orthogonally. (C) Conceptual schematic combining visibly/thermally camouflaged patch and pattern indication process based on modulation of the polarization angle. (D) Photo image of SANCs on Au attached at the surface of temperature-variable silvery object. Scale bar is 1 cm. (E) Thermal image of hidden state with the emissivity matched between the surface and SANCs under unpolarized light. (F) Thermal image of the identified state based on application of linear polarization.

In recent years, everlasting surveillance and reconnaissance by sophisticated detectors have hindered camouflage. Among them, in thermal camouflage systems, variations in the background temperature hinder disclosure.^50^^,^^51^^,^^52^^,^^53^ Hence, there have been various approaches to developing an adaptive thermal camouflage system by tuning the emissivity of the target object. However, the time-consuming operating processes and high energy losses for tuning/sustaining thermal emissions have obstructed their practical operation. Figure 5A shows a conceptual view of the polarization-driven camouflage patch, which shows fine tunability by a simple operation process, *i*.*e*., polarizer rotation (Figures S8 and S9). Therefore, under p-polarization, the patches exhibit a higher emissivity than the s-polarization state. Based on this concept, Figure 5B shows the capability of multifunctional encryption patches in that the SANCs are orthogonally placed. Therefore, under unpolarized light, all SANCs exhibit the same emissivity, whereas under s-polarized or p-polarized light, they have different emissivities corresponding to each position in the pre-arranged direction. Based on the basic properties of SANCs, we developed an adaptive camouflage patch with an encrypted thermal imaging pattern for a temperature-variable object. Owing to their similar color reflections under all intermediate phases and polarizations, SANCs show covert ability on the silvery object surface (Figure S8). Figure 5C shows a conceptual schematic of the multispectral camouflage patch. As revealed in the scheme, SANCs show multispectral characteristic with gray-tone outward and tunable thermal emissive properties. For experimental confirmation, we placed the SANCs orthogonally between the center and surrounding sample. As a result, Figure 5D represents visibly covert pattern (*i*.*e*., the pattern is hidden with regard to the background). In addition, we experimentally confirmed that all the intermediate phases represent a silvery color. Meanwhile, in the infrared range, they exhibit the same thermal emission; on the other hand, at the polarization angle (p-polarization), each position (*i*.*e*., center and surrounding SANCs) shows different apparent temperatures by ∼12%, owing to large emitted power difference of ∼15% (Figure 3E), unveiling encrypted information.

### Conclusion

In conclusion, we have presented a polarization-dependent infrared emission regulator with fine tunability of the thermal emission without sustained heat energy. Optical simulation confirmed the modulation performance with respect to the geometrical parameters (*i*.*e*., thickness and porosity), providing a design rule for a wide tuning range. We verified the fine tunability of the fabricated SANCs over polarization through thermal infrared imaging. In addition, the measured emissivity spectra and thermal analysis exhibited stable uniformity of fabrication owing to the well-matched tendency with the simulation result. For a practical demonstration of the fine control of emissivity, we demonstrated an active thermal camouflage patch using several fabricated samples, some of which were differently aligned with the optical axis. The designed patch hides the pattern in an unpolarized state while revealing the information at a specific polarization, which manifests its ability for thermal encryption. The possibility of dynamic modulation of the emitter is important. Based on diverse dynamic polarization modulators, we believe our suggested structure will show pragmatic applicability via integrating liquid crystal and motorized polarizer.^15^^,^^54^ Moreover, our SANCs can be formed using other chalcogenide PCM (*e*.*g*., Ge-Sb-Se-Te, Sb_2_S_3_, and Sb_2_Se_2_), which exhibits a large complex refractive index variation. Given the versatility and fine tunability of SANCs, the proposed emission regulator could be applied in multimodal anti-counterfeit and heat management systems.

### Limitations of the study

In addition to GST, any chalcogenide PCM, such as Sb_2_S_3_, and In_3_SbTe_2_ can demonstrate SANCs. However, we could not consider all the materials owing to the wide variety of chalcogenide phase change materials. Meanwhile, the proposed SANCs exhibit sufficient modulation capability to perform active thermal camouflage over the polarization angle variation. In this study, we demonstrated thermal encryption for a representative application: the fighter plane at a specific background temperature. For practical cases, the thermal measurement of SANCs under dynamic ambient temperature and different objects is required, which should be addressed in future studies.

## STAR★Methods

### Key resources table

**Table:** 

REAGENT or RESOURCE	SOURCE	IDENTIFIER
**Software and algorithms**		

Origin 2022	Origin lab	https://www.originlab.com/
MATLAB R2013b	Mathworks	https://www.mathworks.com/
RSoft	Synopsys	https://www.synopsys.com/

**Other**		

E-beam evaporator	Korea Vacuum Tech	http://www.koreavac.com/index.php

### Resource availability

#### Lead contact

Further information and requests for resources and reagents should be directed to and will be fulfilled by the Lead Contact, Young Min Song at ymsong@gist.ac.kr.

#### Materials availability

This study did not generate new unique reagents.

### Method details

#### Optical simulation

A rigorous coupled-wave analysis (RCWA) method was employed to calculate the emissivity of the SANCs using commercial software (DiffractMOD, RSoft Design Group, USA). The second diffraction order and a grid size of 0.1 nm-square grid size were utilized during the optical simulation to numerically calculate the stable emissivity. Material dispersions and extinction coefficients were considered for the entire simulation process to obtain accurate spectral results. The complex refractive indices of a-GST/c-GST were obtained from the literature.^55^ Commercial MATLAB software (Mathworks, USA) was also used to calculate the effective complex refractive indices based on the volume average theory.^56^

#### Optical characterization

The emissivity spectra of the fabricated samples in the infrared region were characterized by measuring their reflectance spectra using a Fourier transform infrared spectrometer (Spectrum Paragon, Perkin Elmer, Inc., USA) at a near-normal incidence (10°) to avoid the reflection to the light source. The emissivity barely varies by tilting the sample to 10° (Figure S10). All measurements were calibrated using Au as the standard. Because infrared rays cannot pass through the gold substrate, emissivity spectra were derived from the measured reflectance spectra (*i*.*e*., E = 1 – R). Infrared images were captured using a thermal camera (FLIR E6-XT; FLIR, USA) (Figure S9). To stabilize the measurement environment, we sufficiently warmed up the SANCs on the hotplate (over 10 min) and mounted an antireflective IR focusing lens (LA9410-E3, THORLABS, USA) to improve image quality. SEM (S-4700, Hitachi Hi-Technologies, Japan) was used to observe the cross section of the fabricated SANCs.

#### Deposition of polarization-driven emission regulator with SANCs

The SANCs were fabricated using GLAD to achieve a tilted nanocolumnar structure. For rigid samples, single-side polished silicon (100) wafers were used as the substrate and were treated with a buffered oxide etchant for 3 min to remove the native oxide layer. The substrate was sequentially sonicated for 5 min in acetone, methanol, and deionized (DI) water to remove impurities. Both the metal (Au) and SANCs (GST) were deposited by electron beam evaporation (KVE-E2000, Korea Vacuum Tech Ltd, Korea) under a high vacuum (∼10^−6^ Torr). The Au film was deposited at a rate of ∼2 Å s^−1^ to a thickness of 100 nm that was sufficient to form a metal reflector. SANCs were deposited on the Au film after mounting the substrate on an inclined sample holder (customized) to the targeted thickness at a rate of ∼1 Å s^−1^. To ensure uniformity, deposition was performed to half of the target thickness; post interruption, the sample was reloaded upside down over the tilted sample holder (after rotation through 180°), and the deposition was resumed.^57^^,^^58^^,^^59^ At this point, the tilted sample holder was maintained facing the GST source in the same direction as the first deposition to form unidirectional slanted nanocolumns.

#### Emitted power equation

The emitted power equation is the integral of the object emissivity and blackbody radiance, as follows:^40^

$$P_{thermal}\left(T_{sample}\right)=\int_{\lambda =7}^{\lambda =13}\varepsilon_{obj}\left(\lambda \right)\times {\;I}_{BB}\left(T,\lambda \right)d\lambda \text{,}$$

where $P_{thermal}$ is the power emitted by the object detected by the IR camera, $\varepsilon_{obj}$ is the emissivity of the object, and $I_{BB}=\left(2\pi hc^{2}/\lambda^{5}\right)/\left[e^{hc/\lambda k_{B}T}-1\right]$ is the spectral radiance of a blackbody at temperature T, where h, c, k_B_, and λ are the Planck constant, velocity of light, Boltzmann constant, and wavelength, respectively. For considering hemispherical emission, the emitted power equation should be integral of the incident angles, as follows:

$$P_{thermal}\left(T_{sample}\right)=2\pi \int_{\theta =0{}^{\circ}}^{\theta =60{}^{\circ}}\;\sin\;\theta \;\cos\;\theta \;d\theta \int_{\lambda =7\mu m}^{\lambda =13\mu m}\varepsilon_{obj}\left(\lambda ,\theta \right)\times {\;I}_{BB}\left(T,\lambda \right)d\lambda \text{,}$$

where θ is the incident angle to the sample. In this paper, we simply calculated emitted power by considering only normal incidence because the emitted power, considering hemispherical emission hardly presents a notable difference (Figure S7).

## Data Availability

All data reported in this paper will be shared by the lead contact upon request. This article does not report original code. Any additional information required to reanalyze the data reported in this article is available from the lead contact on request.
