# Supplementary material for: Polarization-driven thermal emission regulator based on self-aligned GST nanocolumns
Source: iScience. 2022 Dec 9;26(1):105780. doi: 10.1016/j.isci.2022.105780 (PMC9800319; doi:10.1016/j.isci.2022.105780)
Supplement: Document S1. Figures S1–S10 [file mmc1.pdf]

iScience, Volume *26*

Supplemental information

Polarization-driven thermal emission regulator based on self-aligned GST nanocolumns

Joo Hwan Ko, Do Hyeon Kim, Sung-Hoon Hong, Sun-Kyung Kim, and Young Min Song

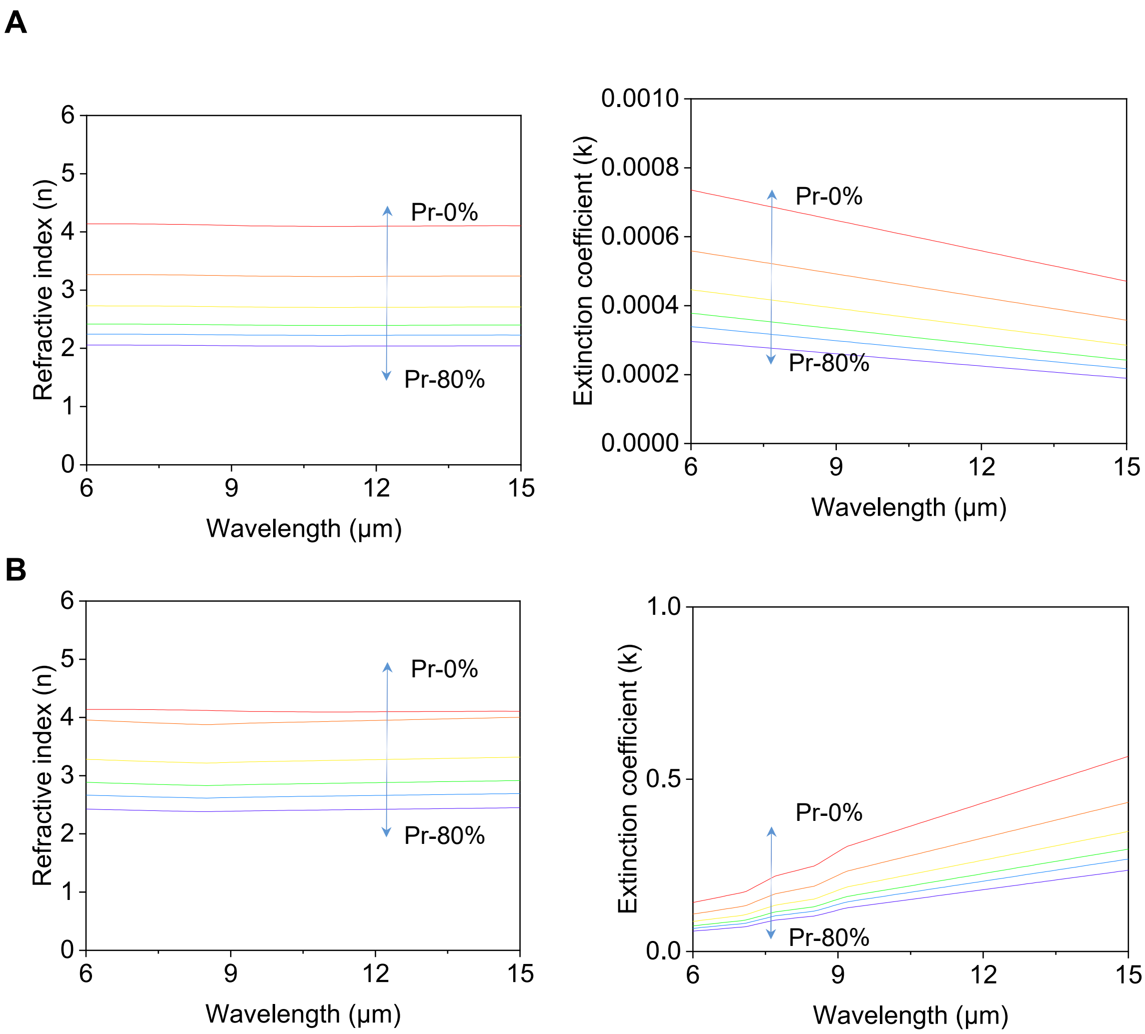

Figure S1, related to Figure 1: complex refractive index of porous (*P_r*) GST

(A-B) Complex refractive index of (A) amorphous-GST (a-GST) and (B) crystalline-GST (c-GST) for different porosity.

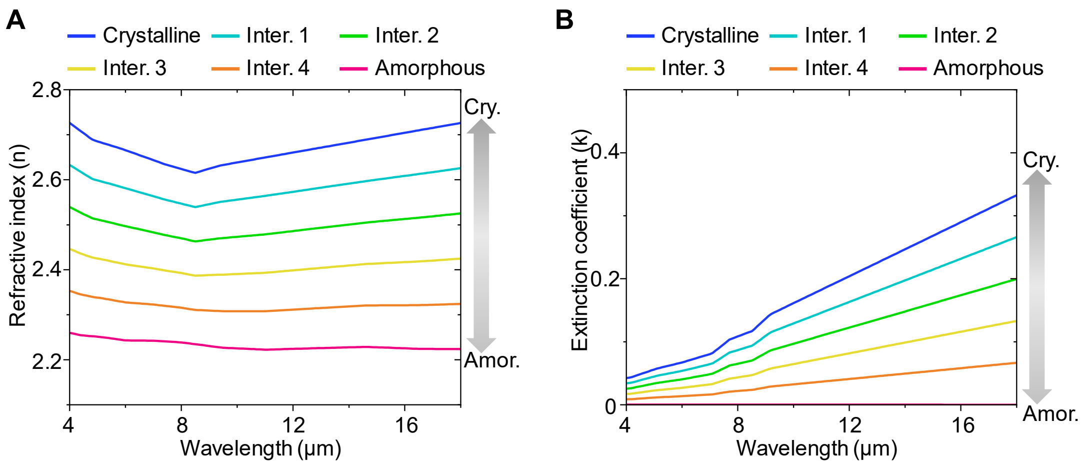

Figure S2, related to Figure 1: The optical constants of SANCs for different phases

(A-B) (A)The refractive indices and (B) extinction coefficients of SANCs with different phases between crystalline and amorphous states.

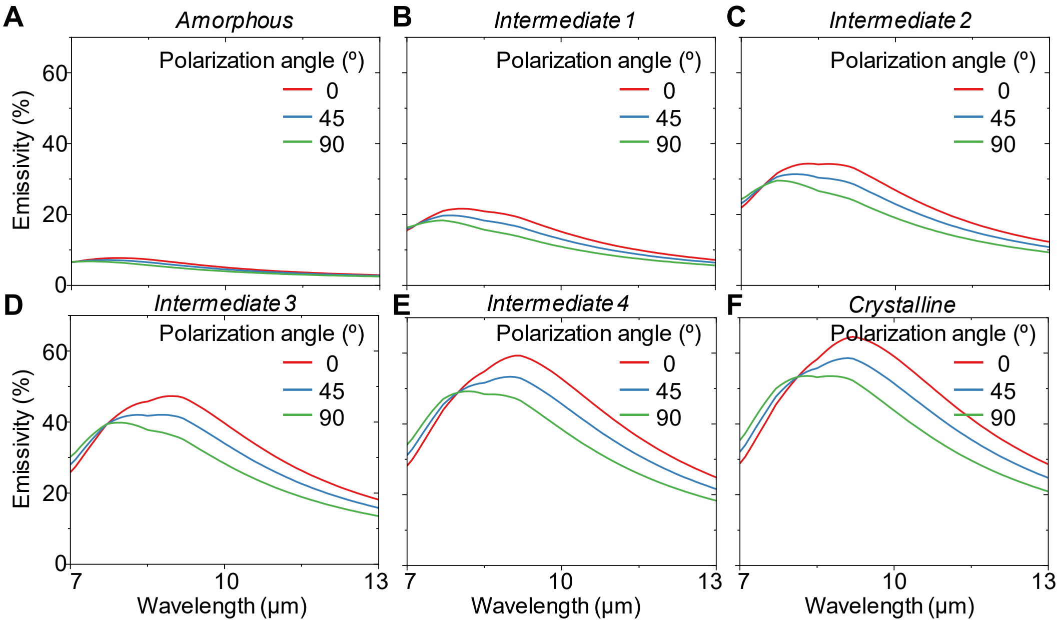

Figure S3, related to Figure 1: The emissivity tunability with different phase

(A-F) The calculated emissivity of SANCs as a function of polarization angles at (A) amorphous, (B) intermediate 1, (C) intermediate 2, (D) intermediate 3, (E) intermediate 4, and (F) Crystalline state.

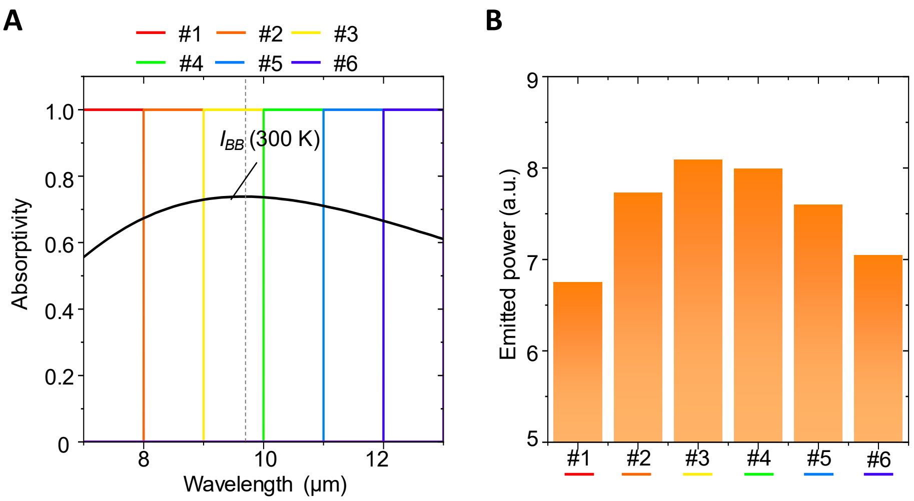

Figure S4, related to Figure 3: The relation between emitted power and the peak of emissivity

(A-B) (A)The refractive indices and (B) extinction coefficients of SANCs with different phases between crystalline and amorphous states.

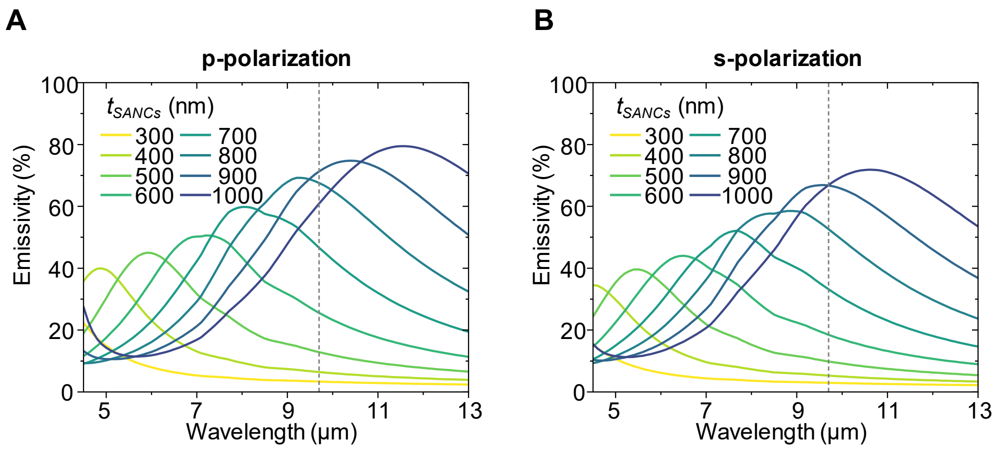

Figure S5, related to Figure 3: Calculated emissivity in terms of thickness and polarization

(A-B) The calculated emissivity of crystalline SANCs as a function of SANCs thickness (300-1000 nm) with (A) p-polarized light and (B) s-polarized light.

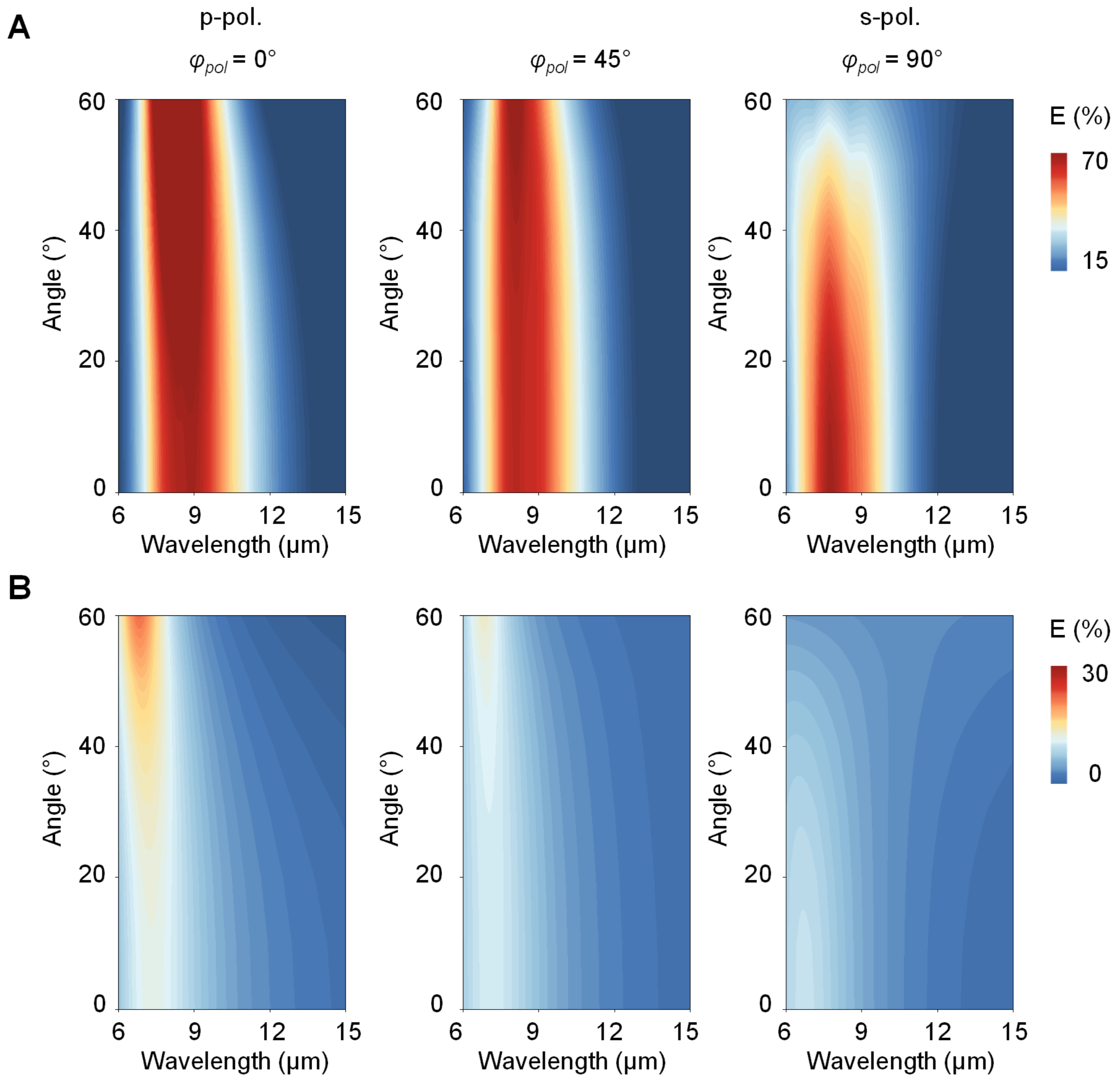

Figure S6, related to Figure 3: Angle dependency of emission corresponding to polarization angle

(A-B) Angle dependency of emission power for (A) c-GST and (B) a-GST

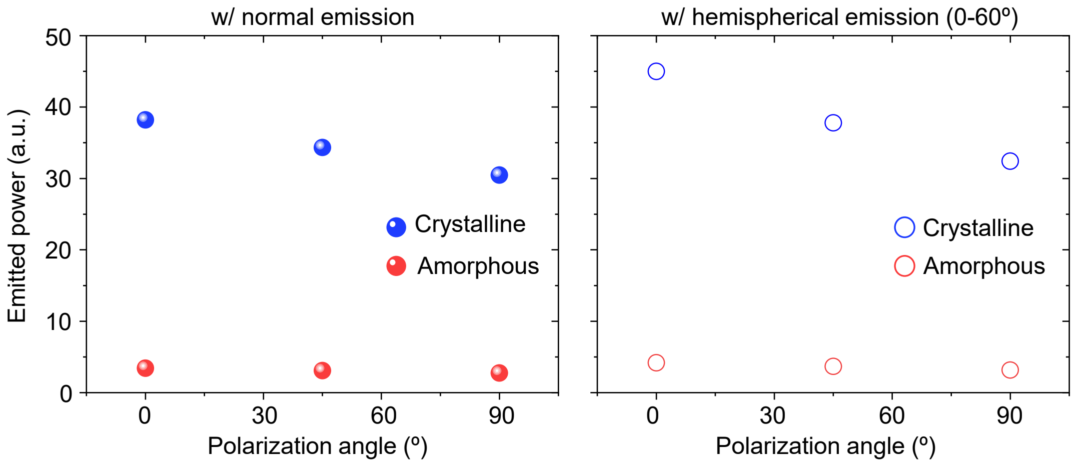

Figure S7, related to Figure 3: Emitted power considering normal and hemispherical emission

Calculated emitted power with normal emission (left) and hemispherical emission from 0-60º (right).

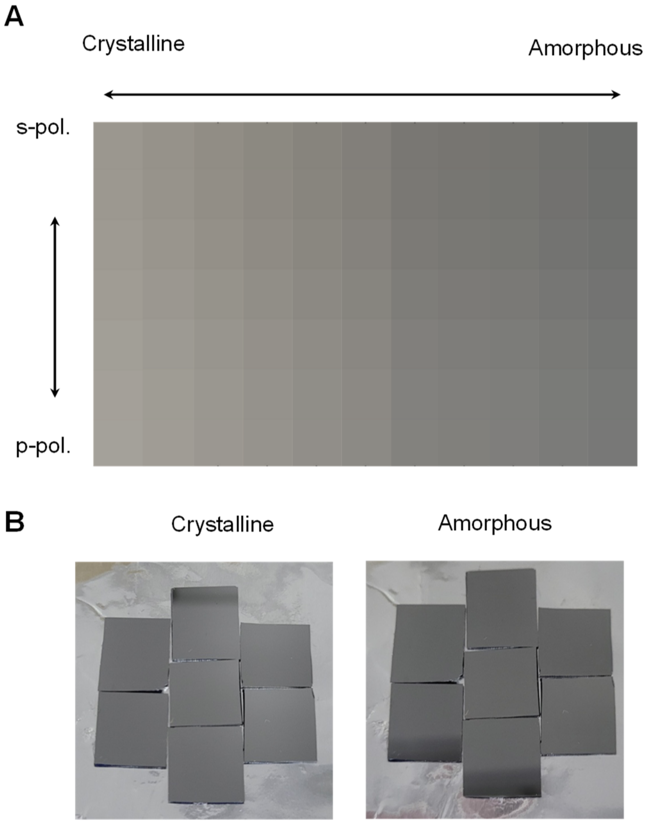

Figure S8, related to Figure 5: Calculated/measured color expression of SANCs on Au

(A) Calculated reflected color of SANCs with varying polarization and phase. (B) Experimentally fabricated SANCs of crystalline/amorphous phase under un-polarized light. Scale bar is 1 cm.

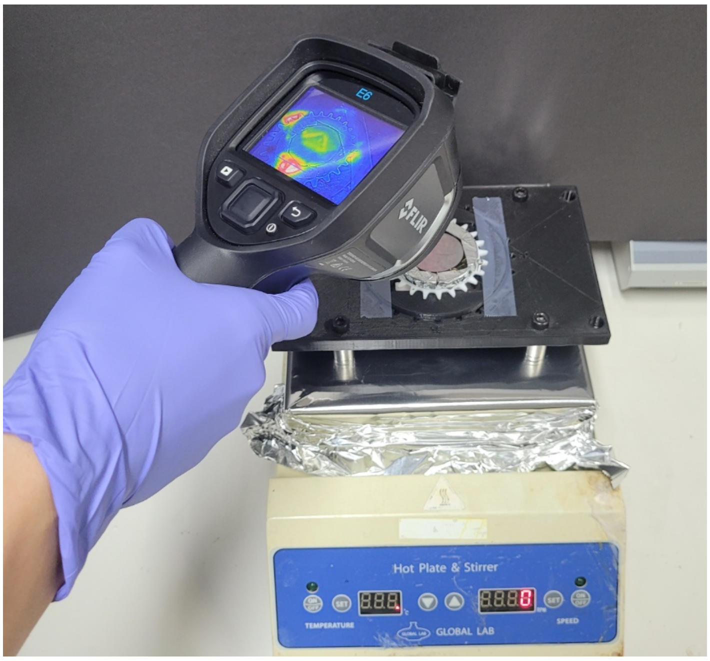

Figure S9 related to STAR METHOD: Measurement setup for thermal imaging of fabricated SANCs

The image of thermal imaging measurement for SANCs under the commercial IR polarizer. The hot plate temperature was set to 100 ºC.

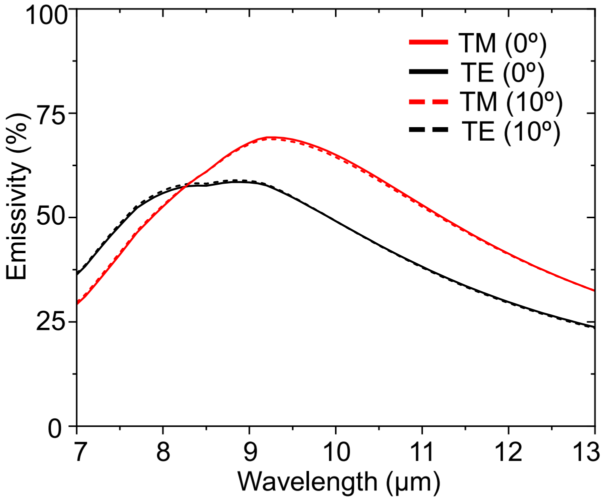

Figure S10, related to STAR ★ METHODS: Incident angle effect on emissivity

Calculated emissivity with different incident angles (0 and 10 º).
